# A Treatise on Surgery

**Published:** 1906-08

**Authors:** 


A TREATISE ON SURGERY. In two volumes. By George R. Fowler, M.D.; Examiner in Surgery, Board of Medical Examiners of the Regents of the University of the State of New York; Emeritus Professor of Surgery in the New York Polyclinic, etc. Two imperial octavos of 725 pages each, with 888 text illustrations and 4 colored plates, all original. Philadelphia and London: W. B. Saunders Company, 1906. Per set: Cloth, $15.00 net; half morocco, $17.00 net.

The second volume of this treatise on surgery, as was to be expected from the first volume, is excellent, and represents the best we have in modern surgery. It covers the surgery, the dorsal and lumbar vertebrae, surgery of the abdominal and pelvic regions, that of the female pelvic organs, and the upper and lower extremities. The essential features are clearly presented and comprise all the recent advancement made in the field of surgery.

There are many first-class illustrations that greatly assist in understanding the operative procedures described.

The newer methods of dealing with hypertrophy of the prostate are briefly, though very satisfactorily, presented.

